# Gasoline particle filter reduces oxidative DNA damage in bronchial epithelial cells after whole gasoline exhaust exposure *in vitro*

**DOI:** 10.1038/s41598-018-20736-z

**Published:** 2018-02-02

**Authors:** Jakob Usemann, Michèle Roth, Christoph Bisig, Pierre Comte, Jan Czerwinski, Andreas C. R. Mayer, Philipp Latzin, Loretta Müller

**Affiliations:** 1 University Children’s Hospital Basel, Basel, Switzerland; 2 Pediatric Respiratory Medicine, Department of Pediatrics, Inselspital, Bern University Hospital, University of Bern, Bern, Switzerland; 3 Adolphe Merkle Institute, University of Fribourg, Fribourg, Switzerland; 4 Laboratory for Exhaust Emission Control, Bern University of Applied Sciences, Biel-Bienne, Switzerland; 5 Technik Thermische Maschinen, Niederrohrdorf, Switzerland

## Abstract

A substantial amount of traffic-related particle emissions is released by gasoline cars, since most diesel cars are now equipped with particle filters that reduce particle emissions. Little is known about adverse health effects of gasoline particles, and particularly, whether a gasoline particle filter (GPF) influences the toxicity of gasoline exhaust emissions. We drove a dynamic test cycle with a gasoline car and studied the effect of a GPF on exhaust composition and airway toxicity. We exposed human bronchial epithelial cells (ECs) for 6 hours, and compared results with and without GPF. Two hours later, primary human natural killer cells (NKs) were added to ECs to form cocultures, while some ECs were grown as monocultures. The following day, cells were analyzed for cytotoxicity, cell surface receptor expression, intracellular markers, oxidative DNA damage, gene expression, and oxidative stress. The particle amount was significantly reduced due to GPF application. While most biological endpoints did not differ, oxidative DNA damage was significantly reduced in EC monocultures exposed to GPF compared to reference exhaust. Our findings indicate that a GPF has beneficial effects on exhaust composition and airway toxicity. Further studies are needed to assess long-term effects, also in other cell types of the lung.

## Introduction

It is well known that air pollution, and especially inhalable particulate matter, is responsible for adverse health effects, including cardiopulmonary diseases^1–3^. One of the main sources of particulate matter is traffic emissions^4,5^. Diesel car exhaust was one of the main contributors to traffic-related particulate air pollution until diesel particle filters were widely applied. The use of a diesel particle filter reduces the emitted particle amount to nearly zero^6^ and, as a result, particulate emissions of gasoline passenger cars are now considered a more important particle source^7^. Furthermore, the traditional port-fuel injection vehicles (fuel injected into the valves) are being increasingly replaced by gasoline direct injection (GDI) cars (fuel injected directly into the combustion chamber)^8^. It is advantageous that GDI cars are more fuel efficient and emit less nitrogen oxide (NO_x_), but, compared to the port-fuel injection vehicles, they release more particulate matter, including combustion-derived nanoparticles^9–11^. Nanoparticles can enter the lungs and even cross the air-blood barrier^12–14^, and combustion-derived nanoparticles have been shown to be toxic^15,16^. In western countries, gasoline cars are more frequent than diesel cars^17^. Additionally, the existing diesel cars are mostly equipped with diesel particle filters letting them emit less particulate matter than gasoline cars^18^. Based on that, one can speculate that gasoline exhaust particles will exceed the mass and amount of diesel exhaust particles in the near future. In order to reduce particle amounts from gasoline cars, and potentially emission toxicity, after-treatment systems (e.g. particle filters) are needed. Deduced from diesel particle filters, the first gasoline particle filters (GPF), both coated and uncoated, have been recently developed and proven to dramatically reduce particulate emissions from gasoline engines^19–22^. However, it is still largely unknown whether the application of GPFs reduces the toxicity of gasoline exhaust emissions.

Epithelial cells (ECs) are the main cells in the human airways. They build a tight barrier in order to protect against pathogens and particles entering the human body^23^. They can orchestrate immune responses and interact with numerous other cell types, e.g. natural killer cells (NKs). NKs belong to the innate lymphoid cells and bridge the innate and the adaptive immune response^24^. They are important to fight viral infections and tumor development^25^. After exposure to environmental stressors, such as viral infections, NKs are recruited from the blood and migrate to the exposure sites. This leads to increased abundances of NKs in the nasal cavity and on the apical side of the airways ECs^26^. Both cell types perform crucial biological functions and are primarily exposed to car exhaust. While ECs have been studied extensively with regard to the toxic effects of traffic exhaust^27–30^, not much is known about the impact of air pollution on NKs^31^.

Our aim was to study the impact of a coated GPF on the cell toxic effects of gasoline exhaust emissions in two models of the human airways: (1) in monocultures of bronchial ECs; and (2) in cocultures of ECs and primary NKs. We exposed our cell models at the air-liquid interface to freshly produced whole exhaust emissions from a gasoline passenger car following a dynamic driving cycle in a previously verified exposure system^32^.

## Material and Methods

### Study design

**Figure 1:**
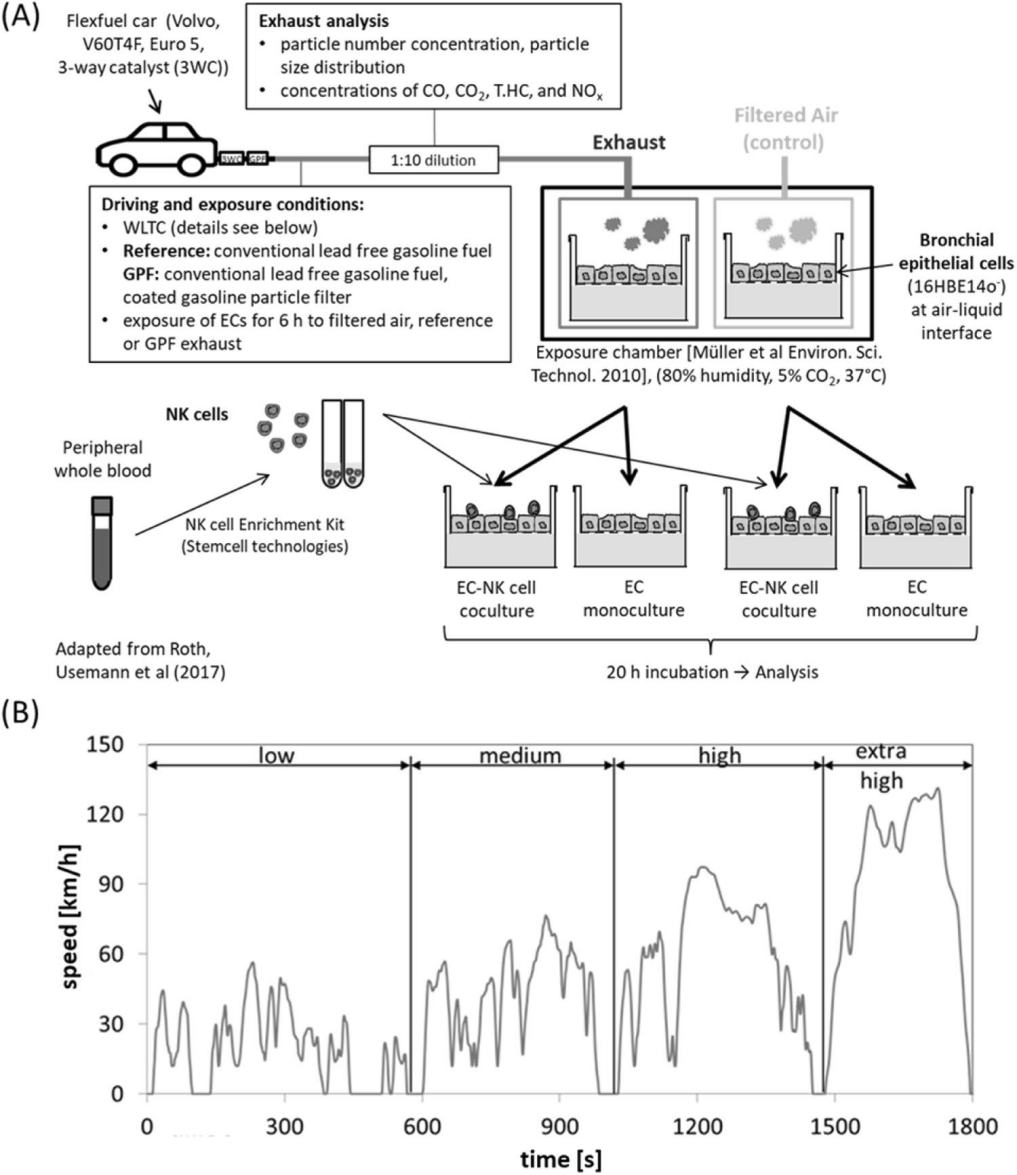
Schematic overview of the study design and driving cycle. **(A)** The Volvo flexfuel car was driven on a chassis dynamometer using conventional lead-free gasoline fuel. ECs were exposed to 1:10 diluted exhaust for 6 h (reference exhaust, GPF cleaned, or filtered ambient air) and subsequently analyzed for immune and cell toxic effects. **(B)** The “worldwide harmonized light-duty vehicles test cycle” (WLTC) represents a standardization of an averaged driving pattern. Figure adapted from^27^. Abbreviations: CO, carbon monoxide; CO_2_, carbon dioxide; GPF, gasoline particle filter; NO_x_, nitrogen oxide; T.HC, total hydrocarbon.

In this experimental *in vitro* study, we compared the impact of the toxic effects of gasoline exhaust emissions between a GDI car with and without a GPF and air control exposure in human bronchial ECs in a monoculture, and in cocultures with NKs. ECs were exposed for 6 h to 1:10 diluted exhaust from a gasoline passenger car driven on a roller dynamometer using a previously established exhaust exposure system^32^, following the dynamic driving cycle “worldwide harmonized light-duty vehicles test cycle” (WLTC) (Fig. 1). Two different cell culture models were studied: (1) EC monocultures, and (2) cocultures of ECs and human, peripheral blood NKs.

### Exposure and exhaust characterization

Details about the exposure protocol have been described previously^27,33^. The only difference in the exposure protocol compared to the published protocol is that, in the current study, we used the WLTC instead of the steady state cycle (SSC) (details in Fig. 1B). The WLTC was developed to closely represent the real-world driving pattern of an average car drive. It is considered to become the standard driving cycle for test procedures worldwide^34^. Briefly, a GDI flexfuel gasoline passenger car was driven following the WLTC using conventional, lead-free gasoline. The car was either driven without (reference) or with a coated GPF (cordierite substrate, 55% porosity, pore size of 14 µm, 200 cells per square inch, coated with palladium and rhodamine), mounted approximately 60 cm downstream of the three-way catalyst. For exhaust characterization, particle number (PN) concentration, carbon monoxide (CO), NO_x_, and total hydrocarbon (T.HC) were analyzed by the Laboratory for IC-Engines and Exhaust Emission Control at the Bern University of Applied Sciences, as described previously^27,28^. The particle size distribution was measured with a scanning mobility particle sizer consisting of a differential mobility analyzer (TSI, DMA model 3081 (long tube)) and a condensation particle counter (TSI, model 3772).

### Cells cultures

The human bronchial EC line 16HBE14o^-^ was grown on transwells until confluence and exposed for 20 h at the air-liquid interface before exposure. We exposed the ECs for 6 h to the exhaust at the Laboratory for IC-Engines and Exhaust Emission Control at the Bern University of Applied Sciences in Biel/Bienne and transported them afterwards to our laboratory at the University Children’s Hospital Basel for incubation and analysis. Since the transport of the ECs took almost two hours, we always added 2.5 × 10^5^ NKs two hours after the exposure to the apical side to half of the exposed ECs to form cocultures. NKs were enriched from the peripheral blood of healthy non-smoking volunteers (age = 30.5 ± 3.7 (mean ± standard deviation), BMI = 21.4 ± 1.9, female/male = 2/2, all Caucasian). The other half of ECs continued to grow as monocultures (Fig. 1). Cocultures and EC monocultures were subsequently incubated for 20 h until harvesting for analysis. The cell model has been validated and described in more detail^27^.

Additionally, we included incubator controls using NKs from the same donor and EC passage number. They were treated similarly to the exposed cells, but were never placed in the exposure system. We compared them to the corresponding air controls and found no differences in cytotoxicity, cell surface receptor expression, intracellular markers, oxidative DNA damage, and oxidative stress (data not shown), indicating no effect from air exposure.

The study was approved by the Ethics Committee Nordwest- und Zentralschweiz, Switzerland. Written informed consent was obtained from all donors.

### Biological analysis and statistics

ECs and NKs were analyzed via flow cytometry for: cytotoxicity, surface expression of EC stress receptors (ULBP2/5/6, ULBP3, MICA/B), chemokine receptor CXCR3 (CD183), activating (CD16, CD314, CD335) and inhibitory (CD158b, CD159a) NK receptors, intracellular NK markers (granzyme B, interferon (IFN) γ, interleukin (IL) 4), and oxidative DNA damage (using the OxyDNA Assay Kit (Calbiochem, MERCK Millipore, Schaffhausen, Switzerland)). The markers chosen for NKs allowed us to reflect on their phenotype and to assess changes in activation and inhibition status, and cytokine production, which are all important for the function of NKs. Gene expression of the EC stress receptors was analyzed via quantitative real-time RT-PCR for mono- and cocultures. Protein release of IL-8 and IP-10 was measured using the Human IL-8 enzyme-linked immunosorbent assay (ELISA) Ready-SET-Go! (Thermo Fisher Scientific, Illkirch Cedex, France) and the Human CXCL10/IP-10 DuoSet ELISA (Bio-Techne, Zug Switzerland) following the manufacturer’s instruction. Samples for the IL-8 ELISA were diluted 1:10, for the IP-10 ELISA we used the samples undiluted. Cellular oxidative stress was assessed via reduced glutathione quantification (Cayman Chemical, Biozol, Eiching, Germany). Cocultures were additionally analyzed for the cell-mediated killing potential of NKs using the 7-AAD/CFSE Cell-mediated Cytotoxicity Assay Kit (Cayman Chemical, Biozol) with the K562 cells as target cells via flow cytometry. All methods have been previously described in detail^27^.

For fluorescence microscopy analysis, cell cultures were fixed with 4% paraformaldehyde for 20 min and stored in phosphate-buffered saline (PBS). After washing (PBS with 1% bovine serum albumin) and permeabilization (0.2% triton X-100 in PBS), the cells were stained with rhodamine phalloidin (1:200 dilution, Molecular Probes) for 45 min and mounted in Vectashield mounting media with DAPI (Reactolab SA). The images were taken on an Olympus BX43 microscope with a XM10 camera and processed with Image J.

Prism GraphPad (Version 6.05, La Jolla, USA) was used for statistical analysis. Data are shown as mean ± standard deviation or [range]. For the comparison between reference and GPF exhaust, all samples were normalized to corresponding air controls. Data from EC monoculture samples from both exhaust exposure conditions were analyzed using the Mann-Whitney test. Since we used the same NK donors for either of the exposure settings, EC coculture and NK samples from different exposure conditions were analyzed using the Wilcoxon Signed Rank test. P < 0.05 was considered statistically significant. The repetition number (N) was 4, unless otherwise stated.

### Data availability

The datasets generated during and/or analyzed during the current study are available from the corresponding author on reasonable request.

## Results

### Exhaust characterization

**Figure 2:**
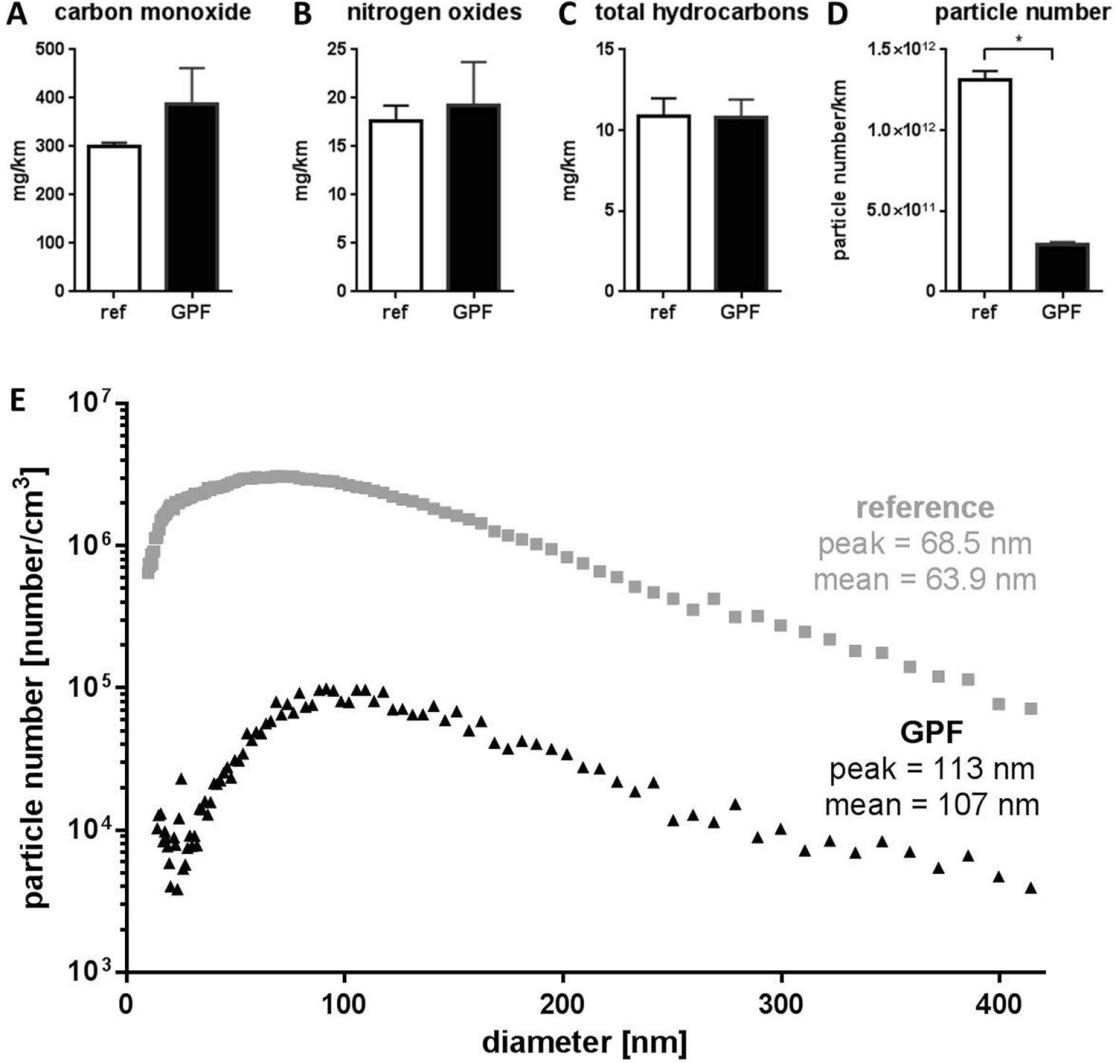
Exhaust characterization. **(A–D)** Data are presented as mean values plus standard deviation, N = 4, *p < 0.05, tested with Mann-Whitney test. **(E)** Particle size distribution measured during SSC. Data are presented as mean values of N = 9.

The measured gaseous pollutants reached similar concentrations for reference and GPF exhaust (Fig. 2A–C). The PN concentration was significantly lower for the GPF exhaust compared to the reference condition (Fig. 2D). The reduction corresponds to a filtration rate of approximately 80%. The mean diameter of the particles increased due to the use of the GPF: under reference condition we observed particles with a mean diameter of 64 nm, and a peak at 69 nm in diameter. Using the GPF resulted in an increased mean diameter of 107 nm and a peak at 113 nm (Fig. 2E). These data were generated while driving a SSC (20 min periods of constant velocities of 95 km/h, 61 km/h, 45 km/h, 26 km/h, or idling), since it is technically impossible to measure the particle size distribution during a dynamic driving cycle with a scanning mobility particle sizer.

### Comparison of immune and cell toxic effects between reference and GPF exhaust exposure

**Figure 3:**
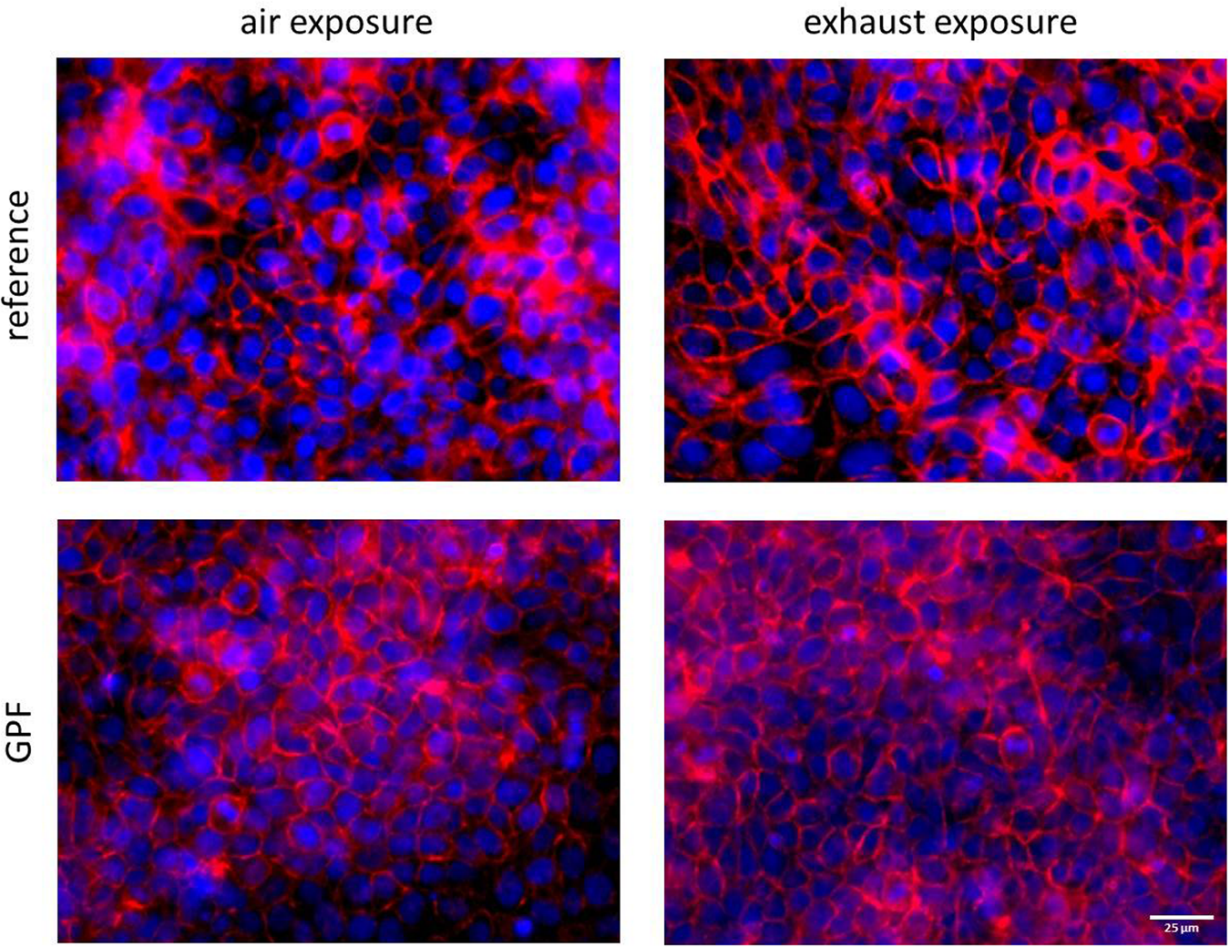
EC monocultures present a monolayer with tight cell-cell contacts. Cell nuclei are shown in blue, F-actin cytoskeleton in red, scale bar = 25 µm.

In order to compare the effects of reference and GPF exhaust exposure directly, we normalized the data to the corresponding air controls. We found no differences in cytotoxicity; neither for ECs in monocultures or cocultures, nor for NKs (Table 1, Table 2). In microscopy analysis, the EC monocultures and cocultures presented a tight, confluent monolayer, no holes were visible and no differences between air and exhaust exposures were found (Fig. 3).

**Table 1:** Comparison of exposure effects between reference and GPF exhaust in EC monocultures and cocultures.

			Reference	GPF	p-value
**Flow cytometry measurement**					
**Cytotoxicity (% dead EC)**		MC	1.04 [1.02–1.06]	1.04 [1.02–1.06]	0.49
		CC	0.96 [0.92–1.02]	0.99 [0.92–1.13]	0.28
**EC surface markers (MFI)**	**MICA/B**	**MC**	1.05 [0.82–1.52]	1.27 [1.06–2.53]	0.49
		**CC**	0.96 [0.45–1.83]	1.56 [0.74–2.59]	0.38
	**ULBP2/5/6**	**MC**	1.21[0.84–1.64]	1.27 [1.03–2.03]	0.69
		**CC**	0.83 [0.63–1.16]	1.25 [0.85–1.69]	0.25
	**ULBP3**	**MC**	1.01 [0.82–1.36]	1.17 [0.98–3.11]	0.49
		**CC**	0.91 [0.48–1.26]	1.23 [0.75–2.00]	0.25
	**CD183**	**MC**	0.93 [0.86–1.00]	097 [0.75–1.50]	>0.99
		**CC**	1.01 [0.72–1.42]	1.06 [0.71–1.11]	>0.99
**DNA damage (MFI)**		**MC**	1.34 [1.24–1.46]	0.85 [0.72–0.94]	*0.03
		**CC**	0.88 [0.68–1.01]	0.97 [0.71–1.05]	0.63
**Quantitative real-time RT-PCR**					
**MICA**		**MC**	0.74 [0.69–1.86]	1.12 [0.76–1.31]	0.37
		**CC**	1.13 [0.86–1.28]	0.83 [0.48–1.21]	0.13
**ULBP2**		**MC**	1.21 [0.90–2.36]	1.21 [1.09–1.33]	>0.99
		**CC**	1.20 [1.12–1.27]	1.08 [0.74–1.13]	0.25
**ULBP3**		**MC**	1.12 [0.62–1.47]	1.14 [0.86–1.51]	0.83
		**CC**	1.15 [0.88–1.36]	0.84 [0.55–0.93]	0.25
**IL-8**		**MC**	1.22 [0.87–2.39]	1.00 [0.80–1.66]	0.68
		**CC**	1.39 [1.12–1.89]	1.01 [0.73–1.12]	0.88
**IP-10**		**MC**	1.39 [1.01–2.14]	1.08 [0.47–1.27]	0.34
		**CC**	1.21 [0.94–1.29]	1.47 [1.02–1.73]	0.38
**Protein release (ELISA)**					
**IL-8 (basolateral)**		**MC**	1.22 [1.18–1.38]	1.20 [1.10–1.96]	>0.99
		^**£**^ **CC**	0.78 [0.59–0.89]	1.00 [0.79–1.02]	0.13
**IL-8 (apical)**		**MC**	1.06 [0.94–1.19]	1.45 [0.85–1.81]	0.63
		^**£**^ **CC**	0.65 [0.51–0.85]	0.86 [0.70–1.02]	0.13
**IP-10 (basolateral)**		**MC**	1.18 [1.04–1.31]	0.89 [0.78–1.11]	0.11
		^**£**^ **CC**	0.82 [0.30–1.05]	0.87 [0.75–0.92]	0.63
**IP-10 (apical)**		**MC**	0.98 [0.95–1.52]	0.77 [0.56–1.29]	0.17
		^**£**^ **CC**	0.83 [0.71–0.98]	0.86 [0.64–1.10]	0.63
**Colorimetric assay**					
**Oxidative Stress (GSH/total protein)**		**MC**	1.12 [0.94–1.21]	0.97 [0.85–1.27]	0.69
		^**#**^ **CC**	1.04 [0.87–1.16]	0.96 [0.90–1.15]	0.88

Data are normalized to corresponding air controls (resulting in 1 = no effect). Values are presented as median [range]. ^#^Includes ECs and NKs. ^£^Includes protein released from ECs and NKs. Data from EC monocultures were analyzed by Mann-Whitney test; data from cocultures by Wilcoxon signed-rank test. Abbreviations: CC, coculture; ELISA, enzyme-linked immunosorbent assay; GSH, glutathione; MC, monoculture; MFI, mean fluorescence intensity.

**Table 2:** Comparison of exposure effects between reference and GPF exhaust in NKs cocultured with ECs.

		**Reference**	**GPF**	**p-value**
**Cytotoxicity (% dead NKs)**		0.90 [0.76–1.40]	1.00 [0.97–1.05]	>0.99
**NKs surface markers (MFI)**	**CD16**	0.98 [0.93–1.01]	1.00 [0.93–1.07]	0.25
	**CD158b**	0.98 [0.95–1.15]	1.02 [0.94–1.90]	0.38
	**CD159a**	0.95 [0.87–1.12]	1.00 [0.91–1.27]	0.38
	**CD183**	0.97 [0.86–1.02]	1.08 [0.89–1.20]	0.13
	**CD314**	0.98 [0.96–1.18]	1.03 [0.93–1.45]	0.38
	**CD335**	1.02 [1.01–1.05]	1.00 [0.99–1.06]	0.63
**NKs intracellular markers (MFI)**	**grzB**	1.01 [0.86–1.13]	1.04 [0.92–1.08]	0.88
	**IFN-γ** ^**#**^	0.92 [0.23–1.53]	0.90 [0.82–2.26]	0.88
	**IL-4**	1.07 [0.89–1.47]	0.98 [0.86–1.12]	0.63
**Killing potential (% dead target cells)**		1.03 [0.97–1.10]	0.88 [0.71–1.06]	0.25
**DNA damage (MFI)**		0.89 [0.55–1.10]	0.93 [0.76–1.04]	0.88

Data are normalized to corresponding air controls (resulting in 1 = no effect, ^#^for matter of normalization, negative values were adjusted to positive values by adding the same fixed value to all data points). Values are presented as median [range]. Abbreviations: MFI, mean fluorescence intensity.

**Figure 4:**
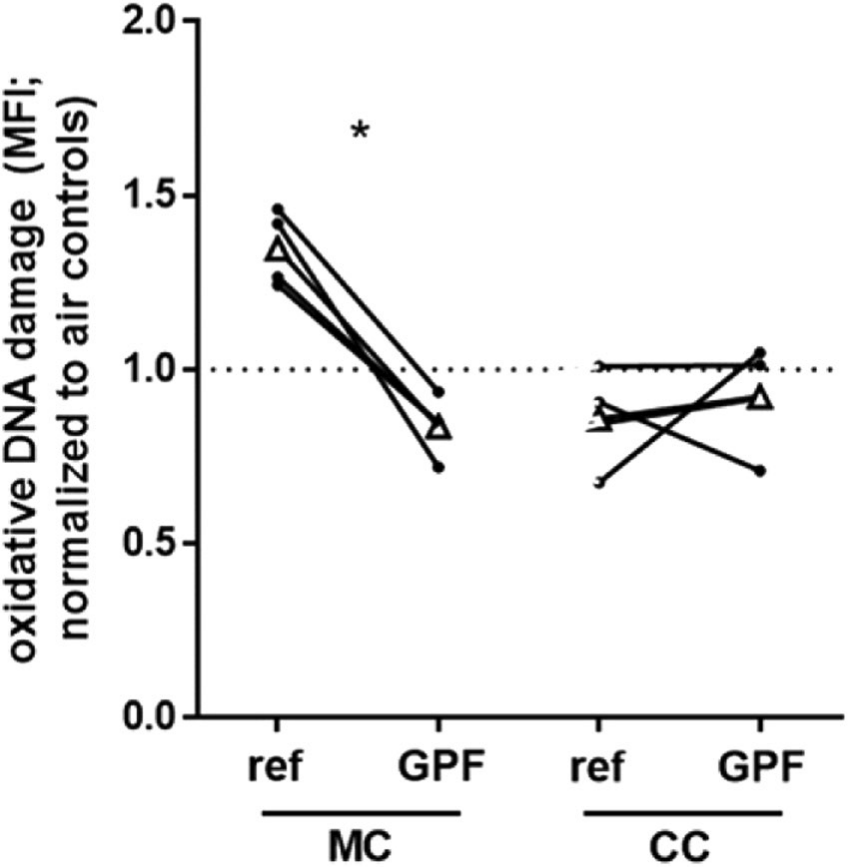
Oxidative DNA damage in ECs exposed to reference and GPF exhaust. Data are presented as single values of MFI. Data points from the same day are connected by a black line. *P < 0.05, MC tested with Mann-Whitney test, CC tested with Wilcoxon signed-rank test. Abbreviations: CC, cocultures; MC, monocultures; MFI, mean fluorescence intensity.

Oxidative DNA damage in EC monocultures was significantly reduced after exposure to GPF compared to reference exhaust (Fig. 4, Table 1). In cocultures, there was no difference in oxidative DNA damage between cells exposed to reference or to GPF exhaust (Table 1, Table 2).

The mRNA of *MICA* in cocultures showed a weak association of reduced levels and exposure to GPF compared to reference exhaust. The gene and surface expression of the other EC stress receptors, and the CD183 and mRNA levels of *IL-8* and *IP-10*, did not differ in cocultures and in monocultures between the exposure to reference and GPF exhaust. The protein levels of IL-8 and IP-10 did not differ between the two exposures, neither in monocultures nor in cocultures (Table 1). There was a weak association of increased CD183 surface expression on NKs and exposure to GPF compared to reference exhaust. The expression of the other NK surface and intracellular markers, and NK killing potential did not differ between the exposure to reference and GPF exhaust (Table 2).

## Discussion

We used a coculture model of human bronchial ECs and NKs to study the effects of a coated GPF on the toxicity of gasoline exhaust emissions *in vitro*. Comparing immune and cell toxic effects between reference (without a GPF) and GPF exhaust, we found significantly less oxidative DNA damage in EC monocultures upon exposure to GPF exhaust. Other endpoints (e.g. cytotoxicity, surface receptor expression, gene expression, and oxidative stress) did not differ significantly between the exposure to reference or GPF exhaust.

### Methodological aspects

The exhaust for this study was produced driving the WLTC, which is an internationally recognized driving cycle. It consists of four different phases with different mean speeds, including acceleration and deceleration, has been developed to closely mimic real-world driving behavior, and will be used as a standard test procedure for car emission tests^34^. The SSC driving cycle was developed to simplify the performance of toxicological exhaust exposure studies. It consists of five phases with constant velocity levels (each with 20 min of one mean speed of the WLTC, plus 20 min of idling). Since the WLTC resembles real-word driving more closely; we decided to utilize this driving cycle. Another strength of our study is the exposure to freshly produced whole gasoline exhaust, which consists of particulate and gaseous components. This is important since both components of exhaust emissions have been shown to cause adverse health effects *in vivo* and *in vitro*^1,3,35,36^. Additionally, our exposure system was controlled for temperature, humidity, and CO_2_ concentration in order to achieve optimal cultivation conditions for the cells. The exposure setting included air controls, which were used to normalize the effects of exhaust exposure. This enabled us to control for variations between exposure days, the cell line passages and the NKs from different donors. We used two different cell models: ECs as monocultures and ECs with NKs as cocultures. In order to avoid translation difficulties from animal data to humans, we used cells of human origin; the EC line 16HBE4o^−^ derived from human bronchial ECs, and primary NKs enriched from human peripheral blood. While the impact of air pollution exposure on NKs has not yet been investigated in detail, 16HBE14o^-^ cells have been widely used to study the effects of air pollution on the airways^30,37–39^. Making use of two cell types, and also combining them as cocultures, allowed us to more closely mimic the human airways^40,41^ and to take into consideration part of their complexity^23^. There are many other cell types present in the human airways (e.g. macrophages), for which it would have been of interest to study the effect of exhaust, as well. However, including other cell types in this study was beyond the scope of this project. NKs were added to the previously exposed ECs two hours after the exposure. This procedure lets us mimic the situation *in vivo*: a human body is exposed to exhaust, a stress reaction is induced and ECs attract immune cells, among other NKs. As a consequence, NKs migrate to the apical side of the respiratory epithelium to fight the stressor^42^. Since NKs are not mainly residential in the human airways, but rather get attracted in case of a stress reaction, we decided not to expose NKs directly to the exhaust. However, it is possible that a direct exposure of NKs to exhaust may affect their function. This effect may be weak after exposure to GPF-cleaned gasoline exhaust of this study with only few particles, but effects could be stronger after exposure to exhaust with high particle concentrations as in the reference exhaust of this study or diesel exhaust^31,43^. A recent study exposed human NKs to engineered nanoparticles^44^ or performed an exposure of mice to geogenic dust^45^ and reported an association between higher exposure levels and reduced NK activity. These previous studies indicate that the direct exposure of NKs to different exhaust types may affect NK cell function. Potential effects of unfiltered gasoline exhaust and GPF exhaust on NKs as used in our study was not investigated and should be investigated in future studies.

### Effects of GPF on exhaust emissions

While the analyzed gaseous components of the exhaust were not altered by the use of a GPF, the PN concentration was reduced by 80% compared to the reference condition. This filtration rate, however, is not as high as it can reach with a high quality diesel particle filter (up to 99.9%^6^). The rather low filtration rate of the GPF in our study can be explained by several mechanisms: first, the filter we used, was a prototype and further development is currently underway and may increase the filtration rate. Second, the filter is constructed for direct installation during the initial assembly of the car. However, for our experiments we had to install the filter afterwards in order to compare exposure conditions with and without the GPF. Third, the filter was brand new and the filtration efficiency is known to increase after some use, due to deposits of ash in the filter wall-flow. However, Chan *et al*.^21^ found a very similar reduction (of 68–85%) compared to our study with the use of a coated GPF and utilizing the U.S. Federal Test Procedure (FTP-75), a city driving cycle designed to represent driving conditions in the U.S. The GPF used in that study^21^, and in another study^46^, closely resembles the one we used for our study. It has a cordierite ceramic with a wall-flow design, 50% porosity, 300 cells per square inch, and is coated with a palladium-rhodamine catalyst. The GPF used in our study has a porosity of 55% and 200 cells per square inch and also cordierite ceramic and a palladium-rhodamine coating. While Saffaripour *et al*. found no changes in the particle diameter comparing unfiltered and GPF exhaust^46^, we found a shift towards bigger particles when using the GPF. Given that a scanning mobility particle sizer, as we used it for the measurement of the particle-size distribution, requires minutes to scan the particle number size distribution, we were not able to measure the particle size distribution during the WLTC due to the rapidly changing velocity pattern. Instead, we used data from another project^47^, in which emissions from the same car equipped with or without the same GPF were characterized driving the SSC. While the peak of the particle diameter without GPF was approximately 69 nm, the peak of the particle diameter with GPF was about 113 nm, likely indicating an agglomeration of particles due to the GPF or higher filtration efficiency for smaller particles compared to bigger ones. The slight increase of particle diameter is most probably not biologically relevant, since the particle diameter is still in the range of nanoparticles, which can enter deeply into the lungs^48^.

We applied and tested the GPF as delivered. Since the GPF was delivered as a coated GPF, we also expected to observe oxidative effects on gaseous compounds of the exhaust. This was, however, not the case (no difference was observed in CO and T.HC concentrations). Since the GDI car was also equipped with a three-way catalyst that likely consumed most of the oxygen, it is possible that no further oxidation took place. Additionally, GDI cars emit almost no NO_2,_ and levels of CO and T.HC were considerably low, making it difficult to finally conclude about an effect of GPF coating.

### Biological effects

Our results show a reduction in oxidative DNA damage in ECs after exposure to GPF compared to reference exhaust. To the best of our knowledge, no study to date has investigated the effects of a coated GPF on the toxicity of gasoline exhaust emissions. Studies have investigated the effect of particulate gasoline exhaust components via the removal of exhaust particles using conventional filters not designed for implementation in vehicles^36,49,50^. However, they did not use a GPF designed to be used in passenger cars, and, additionally, they used older gasoline engine technologies. Only one study investigated the effect of an uncoated GPF using modern flexfuel engine technology^22^. They found an increase in antioxidative glutathione response and *IL-8* mRNA expression, and a decrease of the aryl hydrocarbon receptor and genotoxicity after exposure to GPF exhaust compared to reference exhaust. Overall, the use of the uncoated GPF did not completely reduce all toxic effects of the gasoline exhaust^22^.

We believe the reduction in oxidative DNA damage due to the use of the GPF is primarily driven by the decrease in particles released, and increased particle size. The slightly larger size may result in less penetration into the cells and thus less particle interaction with DNA. The GPF may also remove polycyclic aromatic hydrocarbons (PAH) (unpublished data, personal communication) which are known for their genotoxic potential and adhere to the surface of exhaust particles^51^. Given that there is no difference in oxidative stress levels between reference and GPF exhaust exposure, we think the mechanism for the reduction in oxidative damage is not due to the reduction of oxidative stress, but rather directly via the interaction of particles (and PAH on particles) with DNA. Exposure to traffic-related exhaust has been previously associated with increased levels of oxidative DNA damage *in vivo* and *in vitro*^52,53^ and oxidative damage is linked to inflammation and carcinogenesis^54^. Taking our results and this knowledge into consideration, the reduction of oxidative DNA damage due to the use of a GPF may reduce the harmful effects of gasoline exhaust.

Gene expression of the EC stress receptor *MICA* was slightly reduced in ECs of cocultures exposed to GPF compared to reference exhaust. We found no other publication reporting about MICA and particle filters. However, one study showed an increase of *MICA* mRNA levels in ECs after exposure to PM_2.5_^55^. MICA is a surface receptor of ECs which increases as a consequence of cellular stress, e.g. from exposure to oxidant stressors. It also serves as a ligand for activating receptors on the NKs and is of importance in communication between ECs and NKs. Thus, a reduction of MICA may indicate less cellular stress and, in cocultures, may result in less activation of NKs. We did not find any effect of the GPF exhaust emissions in EC monocultures or at the level of surface receptors, suggesting that this finding needs to be confirmed by further studies.

Furthermore, we found a weak association of increased CD183 expression on NKs and exposure to GPF compared to reference exhaust. This effect on NKs may speak to a higher reactivity of the NKs to chemokine signals, which are ligands for CD183^56^. This potentially increased reactivity may facilitate a reaction to alarm signals in the body, and may result in better defense against tumor development or viral infections. However, since we did not find any effects on activating and inhibitory receptors of NKs, this finding would need to be taken with caution and further studies are needed.

The results of the monocultures and cocultures do not show similar trends. One reason maybe a strong coculturing effect, which was previously shown^27^, and which may mask effects of the exhaust exposure. Additionally, a coculture may be better balanced, reflect more closely *in vivo* conditions, and may be better able to tolerate exposure to stressors, such as car exhaust.

Overall, the acute exposure to reference and GPF gasoline exhaust did not induce strong toxicity in our cell models, and, in fact, the toxicity seemed to be much lower compared to diesel exhaust exposure^27,28^. This is in accordance with previous studies showing only minor toxic effects of gasoline exhaust emissions^16,22,27,28^. Gasoline exhaust, however, contains high numbers of particles with metal oxides, so we expected to see an effect of the GPF. We only performed 4 repetitions, which is low and makes it difficult to detect statistically significant effects. However, trends can be seen, and by comparing the reference to the GPF exhaust directly, two contradictory trends (more DNA damage after reference exhaust compared to air, less DNA damage after GPF exhaust compared to air) result in significant changes.

## Conclusion

The reduction in oxidative DNA damage due to the use of a GPF suggests a reduction of the carcinogenic potential of gasoline car exhaust, which would support the application of GPFs. However, these aspects need to be studied in other biological systems, also focusing on chronic effects.
